# 4-Acetylantroquinonol B Inhibits Osteoclastogenesis by Inhibiting the Autophagy Pathway in a Simulated Microgravity Model

**DOI:** 10.3390/ijms21186971

**Published:** 2020-09-22

**Authors:** Chia-Hsin Wu, Ching-Huei Ou, I-Chuan Yen, Shih-Yu Lee

**Affiliations:** 1 Graduate Institute of Aerospace and Undersea Medicine, National Defense Medical Center, Taipei 11490, Taiwan; chiahsin.ch.wu@gmail.com; 2 Department of Anesthesiology, Cheng-Hsin General Hospital, Taipei 11220, Taiwan; o940209@yahoo.com.tw; 3 School of Pharmacy, National Defense Medical Center, Taipei 11490, Taiwan; yenichuan@mail.ndmctsgh.edu.tw

**Keywords:** 4-acetylantroquinonol B, microgravity, osteoclastogenesis, autophagy

## Abstract

Astronauts suffer from 1–2% bone loss per month during space missions. Targeting osteoclast differentiation has been regarded as a promising strategy to prevent osteoporosis in microgravity (μXg). 4-acetylantroquinonol B (4-AAQB), a ubiquinone from *Antrodia cinnamomea*, has shown anti-inflammatory and anti-hepatoma activities. However, the effect of 4-AAQB on μXg-induced osteoclastogenesis remains unclear. In this study, we aimed to explore the mechanistic impact of 4-AAQB on osteoclast formation under μXg conditions. The monocyte/macrophage-like cell line RAW264.7 was exposed to simulated μXg (Rotary Cell Culture System; Synthecon, Houston, TX, USA) for 24 h and then treated with 4-AAQB or alendronate (ALN) and osteoclast differentiation factor receptor activator of nuclear factor kappa-B ligand (RANKL). Osteoclastogenesis, bone resorption activity, and osteoclast differentiation-related signaling pathways were analyzed using tartrate-resistant acid phosphatase (TRAP) staining, actin ring fluorescent staining, bone resorption, and western blotting assays. Based on the results of TRAP staining, actin ring staining, and bone resorption assays, we found that 4-AAQB significantly inhibited μXg-induced osteoclast differentiation. The critical regulators of osteoclast differentiation, including nuclear factor of activated T-cells cytoplasmic 1 (NFATc1), c-Fos, and dendritic cell-specific transmembrane protein (DC-STAMP), were consistently decreased. Meanwhile, osteoclast apoptosis and cell cycle arrest were also observed along with autophagy suppression. Interestingly, the autophagy inhibitors 3-methyladenine (3-MA) and chloroquine (CQ) showed similar effects to 4-AAQB. In conclusion, we suggest that 4-AAQB may serve as a potential agent against μXg-induced osteoclast formation.

## 1. Introduction

In a space environment, several risk factors threaten the health of astronauts, including variations in gravity and radiation [1]. Astronauts face multiple challenges in a microgravity (µXg) environment, including deconditioning of the cardiovascular system, suppression of immune function, and imbalance of bone metabolism during spaceflights [2,3,4,5,6]. Among these, skeletal metabolism-related problems pose a ubiquitous concern among space travelers. One long-term study found that astronauts lose an average bone density of 11% over a 4–6 month mission [2]. In general, astronauts experience decreases of 1% muscle mass and 1.8–2% bone density per month [3]. Although resistance exercises promote muscle mass maintenance, bone loss remains a problem for astronauts due to an imbalance between bone formation and bone resorption. Studies have reported that exposure to a μXg environment results in decreased osteoblast formation [7] and increased osteoclast differentiation [8]. Therefore, targeting osteoclastogenesis has been regarded as a promising strategy to prevent osteoporosis in μXg conditions [8,9].

Osteoclasts originate from the monocyte-macrophage lineage, and many factors are involved in the process of osteoclast differentiation. c-Fos and nuclear factor of activated T-cells cytoplasmic 1 (NFATc1) are two important factors involved in osteoclast differentiation [10,11]. Dendritic cell-specific transmembrane protein (DC-STAMP) also participates in osteoclast differentiation and is related to cell-cell fusion [12,13]. Two inflammatory related pathways, nuclear factor kappa-light-chain-enhancer of activated B cells (NF-κB) and mitogen-activated protein kinases (MAPKs), regulate osteoclast differentiation and function [14]. At present, the methods to alleviate overactivated osteoclasts include inhibiting osteoclastogenesis, inducing cell apoptosis, and inhibiting osteoclast resorption activities [15]. Bisphosphonate, a current treatment for osteoporosis, inhibits osteoclast formation and function by activating the caspase-3 and caspase-9 apoptosis signaling pathways [16,17]. Autophagy is also involved in osteoclastogenesis. Overexpression of Beclin-1 has been observed to suppress osteoclast precursor apoptosis [18] while autophagy inhibitors consistently suppress osteoclastogenesis [19].

Current bone loss prevention methods during space missions involve exercise and drug intervention [20,21,22]; however, a more effective mechanism needs to be developed. 4-acetylantroquinonol B (4-AAQB), a ubiquinone from *Antrodia cinnamomea*, has been found to possess anti-inflammatory [23,24], antioxidant [25], and anti-hepatoma properties [24,26]. A recent study showed that 4-AAQB suppresses autophagic flux through the PI3K signaling pathway in epithelial cancer cells [27]. It is well known that the inflammatory and autophagy signaling pathways are highly associated with osteoclastogenesis [18,28]. These findings imply a role of 4-AAQB in osteoclastogenesis regulation. This study aimed to explore the mechanistic impact of 4-AAQB on osteoclast formation under μXg conditions.

## 2. Materials and Methods

### 2.1. 4-AAQB

4-AAQB was prepared as previously described. Briefly, dried *A. cinnamomea* mycelium was extracted with 95% alcohol, and the extract was successively partitioned with dichloromethane and water. The dichloromethane-soluble fraction (60 g from 1305.3 g of mycelium) was subjected to column chromatography and high-performance liquid chromatography separation as described previously [29,30].

### 2.2. Cell Culture

The RAW264.7 cell line was purchased from the American Type Culture Collection (Manassas, VA, USA). RAW264.7 cells are widely used for studying osteoclastogenesis [9,31,32]. The cells were maintained at 37 °C in an atmosphere of 5% CO_2_ in Dulbecco’s Modified Eagle Medium supplemented with 10% fetal bovine serum. Receptor activator of nuclear factor-κB ligand (RANKL; 100 ng/mL) was added to induce osteoclast differentiation.

### 2.3. Cell Viability Assay

Cell viability was determined using the alamarBlue Cell Viability assay kit (Invitrogen™, Thermo Fisher Scientific Inc., Waltham, MA, USA) as previously described [33]. Briefly, the cells (1 × 10^4^ cells/well) were seeded into 96-well plates for 24 h. After treatment with 4-AAQB or alendronate (ALN) for 72 h, an alamarBlue solution was added for 2 h at 37 °C. The absorbance was measured at 570/630 nm using a spectrophotometer (Spectra Max 190; Molecular Devices, Sunnyvale, CA, USA).

### 2.4. Rotary Cell Culture System (RCCS)

The RCCS (Synthecon, Houston, TX, USA) is a system that provides a low-shear μXg-like environment. The rotary speed of the RCCS was set at 12 rpm, and the cells were incubated in the RCCS for 24 h to simulate μXg (0.004 Xg) or normal-gravity (v-Xg), which rotated around a vertical or horizontal axis [9].

### 2.5. Tartrate-Resistant Acid Phosphatase (Trap) Staining

The cells were fixed with fixation solution supplemented with 10% formaldehyde for 10 min at 25 °C and then stained with TRAP solution (Sigma-Aldrich, St. Louis, MO, USA) for 20 min [34]. Images were visualized with a light microscope (DMi8; Leica Microsystems, Wetzlar, Germany). The number of TRAP (+) cells/well and area of TRAP (+) cells (%) were measured using ImageJ software version 1.50a (National Institutes of Health [NIH], Bethesda, MD, USA).

### 2.6. Actin Ring Immunofluorescence

The cells were fixed with 4% methanol-free formaldehyde for 10 min at 25 °C. Later, the cells were stained with Alexa Fluor^®^ 488 Phalloidin (Cell Signaling Technology, Danvers, MA, USA) in the dark for 15 min at 25 °C. Then, the cells were washed and stained with nuclear 4′,6-diamidino-2-phenylindole dye (Cell Signaling Technology) in the dark for 5 min at 25 °C [35]. Images were visualized with a light microscope (DMi8; Leica Microsystems). The number of actin rings/well was measured using ImageJ software version 1.50a (NIH).

### 2.7. Osteo Assay

After being cultured in Corning Osteo Assay (Corning, Inc., Corning, NY, USA) 96-well plates for 120 h, the cells were incubated with the bleach solution for 5 min at room temperature. The plates were washed with distilled water twice and dried at room temperature for 5 h [36]. Images were visualized with a light microscope (DMi8; Leica Microsystems). The percentage of bone resorption area was measured using ImageJ software version 1.50a (NIH).

### 2.8. Western Blotting

Immunoblotting was performed using primary antibodies against cyclin D3 (BD Biosciences, San Diego, CA, USA), Atg 5, Atg 7, caspase-9, caspase-8, caspase-3, c-Fos, NFATc1, PARP (Cell Signaling Technology), cyclin E1, p21 (GeneTex, Irvine, CA, USA), DC-STAMP (Merck, Kenilworth, NJ, USA), β-actin, LC3B, and p62 (Proteintech, Chicago, IL, USA). The blots were developed with an enhanced chemiluminescence kit (Amersham Biosciences, Buckinghamshire, UK) and measured using a luminescent image analyzer (LAS-3000; Fuji Photo Film Co., Ltd., Tokyo, Japan) [37].

### 2.9. Statistical Analysis

All data are presented as the mean ± standard error of the mean. Significant differences between the means of two groups were determined with a *t*-test using GraphPad Prism software version 7.0 (GraphPad Software, San Diego, CA, USA). Statistical significance was considered at *p* < 0.05.

## 3. Results

### 3.1. 4-AAQB Cytotoxicity under Both v-Xg And µXg Conditions

**Figure 1:**
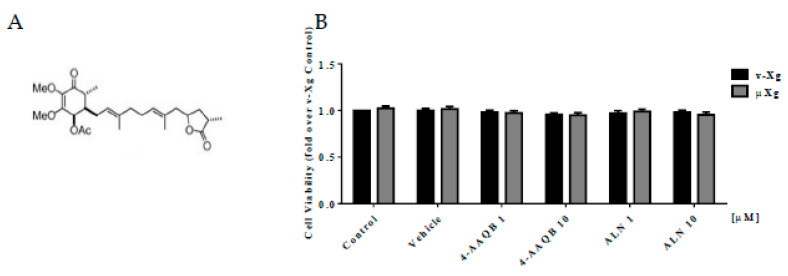
Effects of 4-AAQB on RAW264.7 cell viability. Chemical structure of 4-AAQB (**A**). Cell viability was assayed by alamarBlue for 2 h after cell stimulation with v-Xg or µXg for 24 h (**B**). The quantitative results are presented as the mean ± SEM (*n* = 3).

Figure 1A shows the chemical structure of 4-AAQB. 4-AAQB and ALN, a positive control, did not alter cell viability in either v-Xg or µXg conditions (Figure 1B).

### 3.2. 4-AAQB Effects on RANKL-Induced Osteoclast Formation and Resorption Ability in Both v-Xg and µXg Conditions

**Figure 2:**
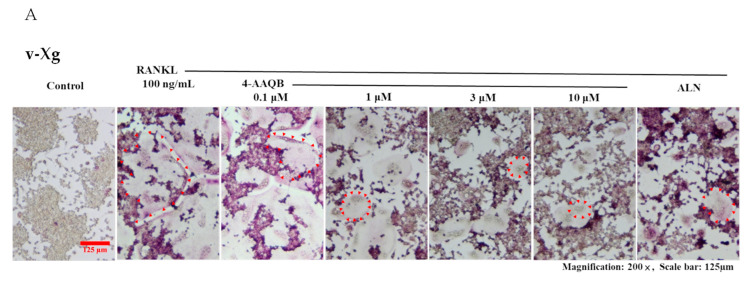

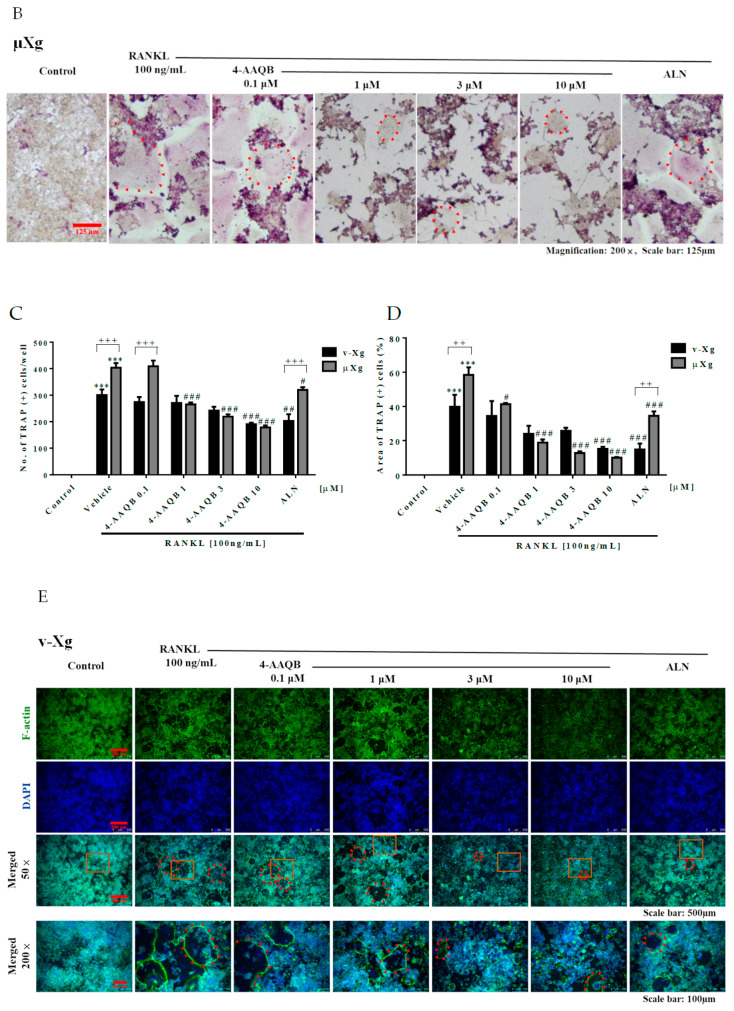

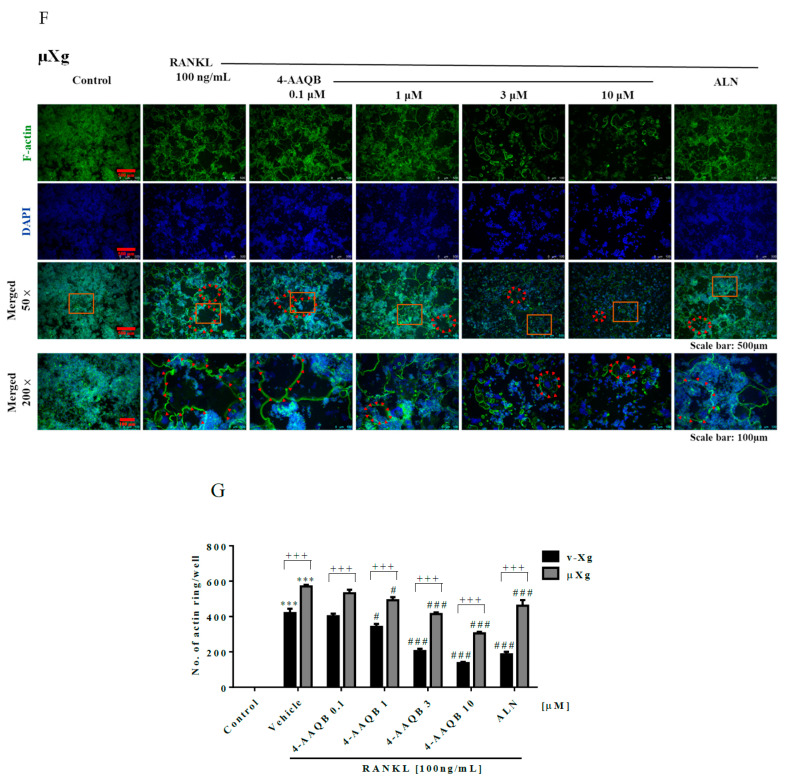
4-AAQB attenuation of osteoclast formation after v-Xg and µXg stimulation. After exposure in v-Xg (**A**) or µXg (**B**) for 24 h, and the cells were treated with 4-AAQB (0.1, 1, 3, or 10 µM) or ALN (10 µM) combined with RANKL for 72 h. The quantitative results of the number of TRAP (+) osteoclasts/well (**C**) and area of TRAP (+) osteoclasts (%) (**D**) represent the mean ± SEM (*n* = 6). In addition, fluorescent staining (actin ring and DAPI; (**E**,**F**)) and quantitative results (number of actin rings/well; (**G**)) were conducted. Osteoclasts or actin rings are indicated by red arrows. Orange rectangles represent the areas magnified 200 times. *** *p* < 0.001 versus control; # *p* < 0.05, ## *p* < 0.01, and ### *p* < 0.001 versus RANKL; ++ *p* < 0.01 and +++ *p* < 0.001 versus v-Xg.

**Figure 3:**
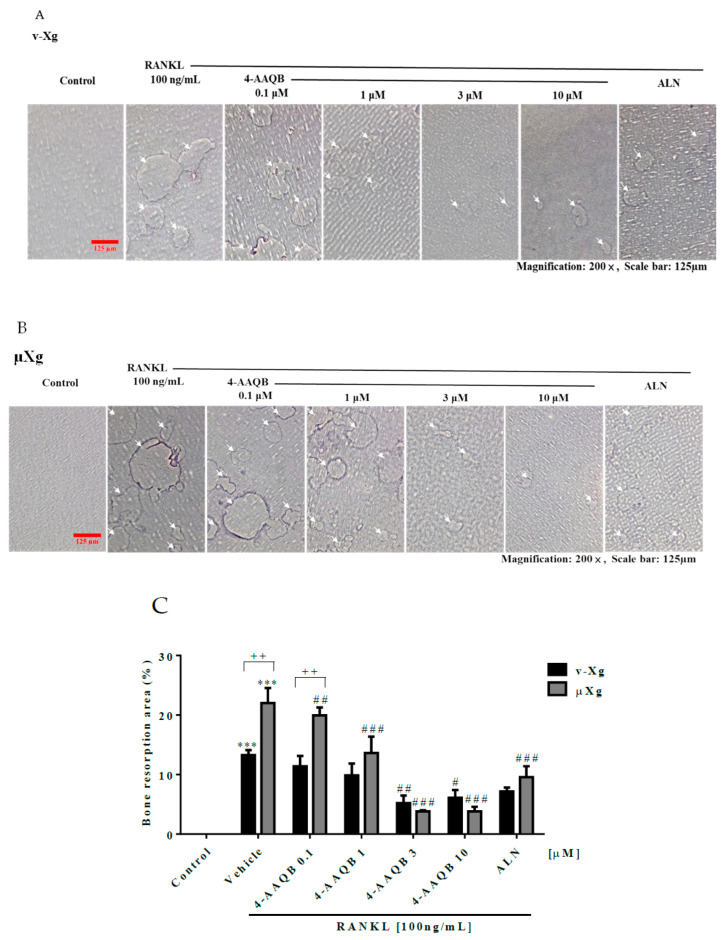
4-AAQB attenuation of osteoclast resorption ability after v-Xg and µXg stimulation. After exposure in v-Xg (**A**) or µXg (**B**) for 24 h, and the cells were cultured in osteo assay 96-well plates and treated with 4-AAQB (0.1, 1, 3, or 10 µM) or ALN (10 µM) combined with RANKL for 120 h. The bone resorption area (%) (**C**) quantitative results represent the mean ± SEM (n = 4). White arrows indicate the resorption pits. *** *p* < 0.001 versus control; # *p* < 0.05, ## *p* < 0.01, and ### *p* < 0.001 versus RANKL; ++ *p* < 0.01 versus v-Xg.

To examine the effects of 4-AAQB on osteoclast formation, TRAP staining was conducted. Our results showed that 4-AAQB eliminated both the number and area of TRAP (+) osteoclasts (Figure 2A–D) under both v-Xg and µXg conditions. The number of actin rings was also consistently decreased by 4-AAQB (Figure 2E–G). We further assessed the effects of 4-AAQB on osteoclast resorption activity using an osteo assay. 4-AAQB significantly decreased osteoclast resorption activity as expected (Figure 3A–C). Based on these findings, 4-AAQB attenuates RANKL-induced osteoclast formation and resorption activity. These findings indicate that 4-AAQB efficiently attenuates osteoclast formation in both v-Xg and µXg conditions. Interestingly, 4-AAQB displayed greater efficacy in µXg than in v-Xg.

### 3.3. Inhibition of Essential Osteoclast Differentiation Pathways by 4-AAQB, Especially in µXg Conditions

**Figure 4:**
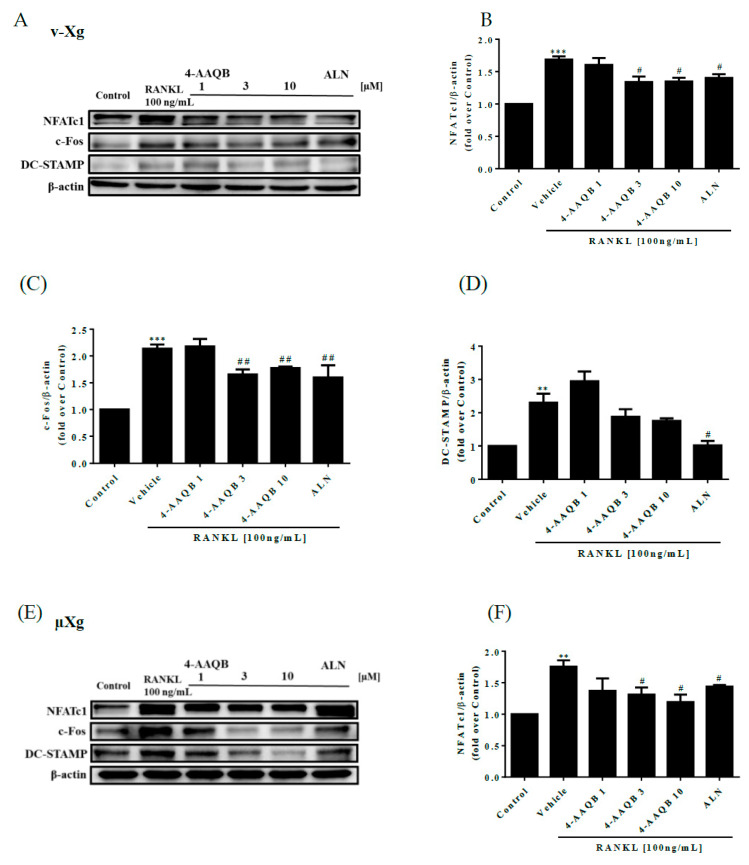

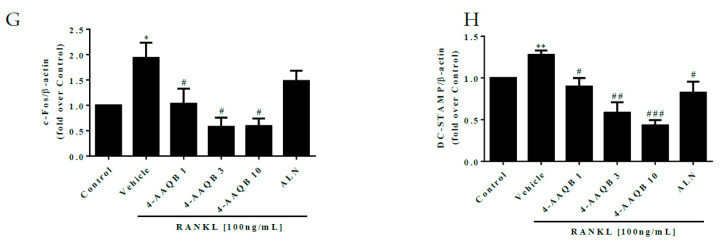
Effects of 4-AAQB on the osteoclastogenesis signaling pathway. Osteoclastogenesis proteins were assayed by western blotting after exposure to v-Xg (**A**) conditions for 24 h. The cells were treated with 4-AAQB (1, 3, or 10 µM) or ALN (10 µM) combined with RANKL for 54 h. The quantitative results of NFATc1 (**B**), c-Fos (**C**), and DC-STAMP (D) represent the mean ± SEM (n = 3). In addition, after exposure to μXg (**E**) conditions for 24 h, the cells were treated under the same stimulation conditions as the v-Xg group for 48 h. The quantitative results (**F**–**H**) represent the mean ± SEM (n = 3). * *p* < 0.05, ^**^p < 0.01 and *** *p* < 0.001 versus control; # *p* < 0.05, ## *p* < 0.01, and ### *p* < 0.001 versus RANKL.

To confirm the inhibitory effects of 4-AAQB on osteoclastogenesis, the protein levels of c-Fos, NFATc1, and DC-STAMP were analyzed by western blotting. We showed that the NFATc1, c-Fos, and DC-STAMP protein levels were decreased by 4-AAQB in both v-Xg (Figure 4A–D) and µXg conditions (Figure 4E–H). Thus, 4-AAQB appears to successfully attenuate the osteoclastogenesis signaling pathway, especially under µXg conditions. In µXg, 4-AAQB not only alleviates osteoclast formation and resorption activities but also suppresses the key regulators of osteoclast differentiation and cell-cell fusion.

### 3.4. 4-AAQB Effects on Osteoclast Apoptosis and Cell Cycle Arrest under μXg Conditions

**Figure 5:**
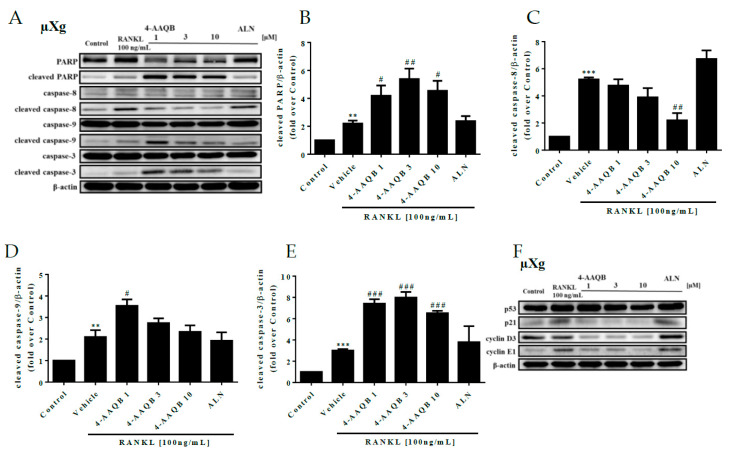

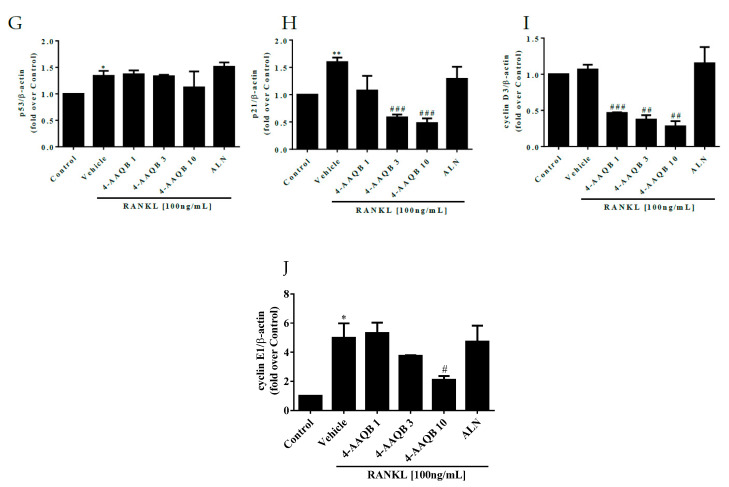
Effects of 4-AAQB on osteoclast apoptosis and the cell cycle after μXg stimulation. After exposure to μXg (**A**) conditions for 24 h, the cells were treated with 4-AAQB or ALN combined with RANKL for 48 h. Apoptotic proteins were assayed by western blotting. The quantitative results of cleaved PARP (**B**), cleaved caspase-8 (**C**), cleaved caspase-9 (**D**), and cleaved caspase-3 (**E**) represent the mean ± SEM (n = 3). In addition, the cell cycle proteins were also assayed by western blotting (**F**). The quantitative results of p53 (**G**), p21 (**H**), cyclin D3 (**I**), and cyclin E1 (**J**) represent the mean ± SEM (n = 3). * *p* < 0.05, ** *p* < 0.01, and *** *p* < 0.001 versus control; # *p* < 0.05, ## *p* < 0.01, and ### *p* < 0.001 versus RANKL.

Cell apoptosis and cell cycle-related proteins were further analyzed under μXg conditions. We found that 4-AAQB increased cleaved caspase-3 and cleaved PARP but decreased cleaved caspase-8 protein levels (Figure 5A–E). Furthermore, the protein levels of p21, cyclin D3, and cyclin E1 were suppressed by 4-AAQB (Figure 5F–J). These findings indicate that 4-AAQB not only promotes cell apoptosis but also causes cell cycle arrest at the G1-S phase in μXg.

### 3.5. 4-AAQB Inhibition of RANKL-induced Osteoclastogenesis through an Autophagy-Dependent Pathway Under μXg Conditions

**Figure 6:**
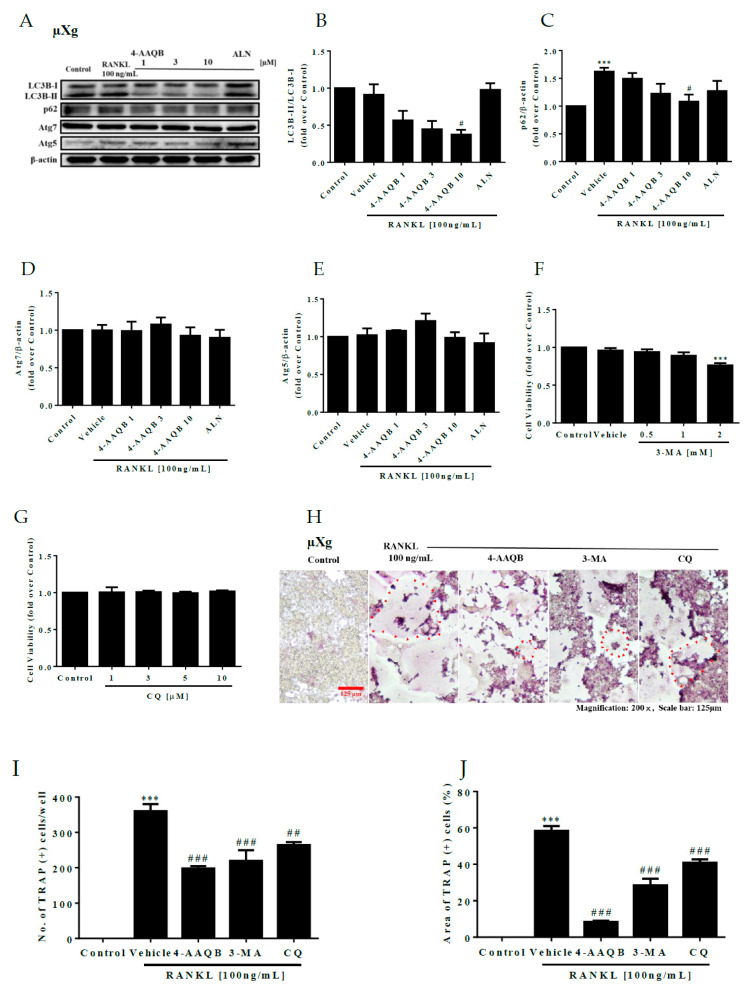

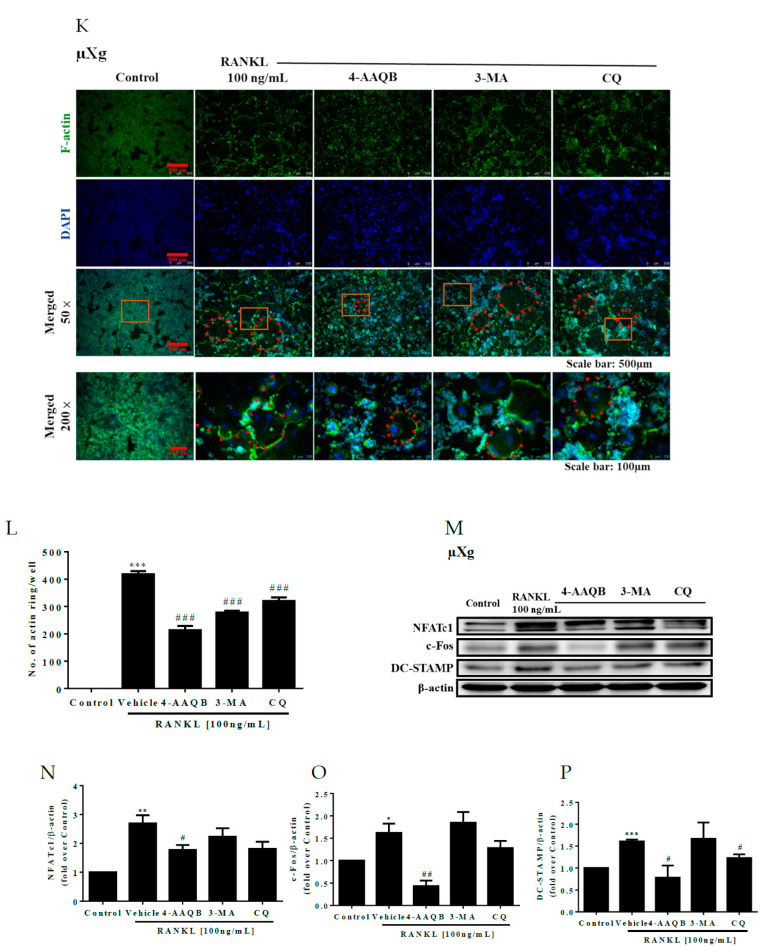
Autophagy inhibitor attenuation of osteoclast formation after µXg stimulation. Autophagy proteins were assayed by western blotting (**A**)**.** The quantitative results of LC3B-II/LC3B-I (**B**), p62 (**C**), Atg 7 (**D**), and Atg 5 (**E**) represent the mean ± SEM (*n* = 3). Cell viability was assayed by alamarBlue for 2 h after cell stimulation under µXg conditions for 24 h; stimulation by 3-MA (**F**) or CQ (**G**) continued for 72 h. The quantitative results represent the mean ± SEM (n = 3). TRAP staining was performed after cell stimulation under µXg (**H**) conditions for 24 h. and the cells treated with 4-AAQB (10 µM), 3-MA (1 mM), or CQ (10 µM) combined with RANKL for 72 h. The quantitative results of the number of TRAP (+) osteoclasts/well (**I**) and area of TRAP (+) osteoclasts (%) (**J**) represent the mean ± SEM (*n* = 6). Fluorescent staining was performed after stimulation under µXg (**K**) conditions for 24 h, and the cells treated with 4-AAQB, 3-MA, or CQ combined with RANKL for 72 h. The quantitative results of the number of actin rings/well (**L**) represent the mean ± SEM (n = 4). After exposure to μXg (**M**) conditions for 24 h, the cells were treated with 4-AAQB, 3-MA, or CQ combined with RANKL for 48 h. Osteoclastogenesis proteins were assayed by western blotting. The quantitative results of NFATc1 (**N**), c-Fos (**O**), and DC-STAMP (**P**) represent the mean ± SEM (n = 3). Osteoclasts or actin rings are indicated by red arrows. Orange rectangles represent the areas magnified 200 times. * *p* < 0.05, ** *p* < 0.01, and *** *p* < 0.001 versus control; # *p* < 0.05, ## *p* < 0.01, and ### *p* < 0.001 versus RANKL.

In addition to cell apoptosis and cell cycle arrest, 4-AAQB significantly decreased the protein levels of LC3B-II/LC3B-I and p62 while minimal change was observed in Atg5 and Atg7 (Figure 6A–E). These findings imply that 4-AAQB regulates osteoclastogenesis through an autophagy-dependent pathway. To confirm this, the autophagy inhibitors 3-MA and chloroquine (CQ) were used in subsequent experiments. The results of TRAP staining (Figure 6H–J) and actin ring immunofluorescence (Figure 6K,L) showed that both 3-MA and CQ attenuated osteoclast formation. Both 3-MA and CQ suppressed osteoclastogenesis-related signaling pathway protein markers (Figure 6M–P). These findings indicate that 4-AAQB inhibits μXg-induced osteoclastogenesis through an autophagy-dependent pathway.

## 4. Discussion

**Figure 7:**
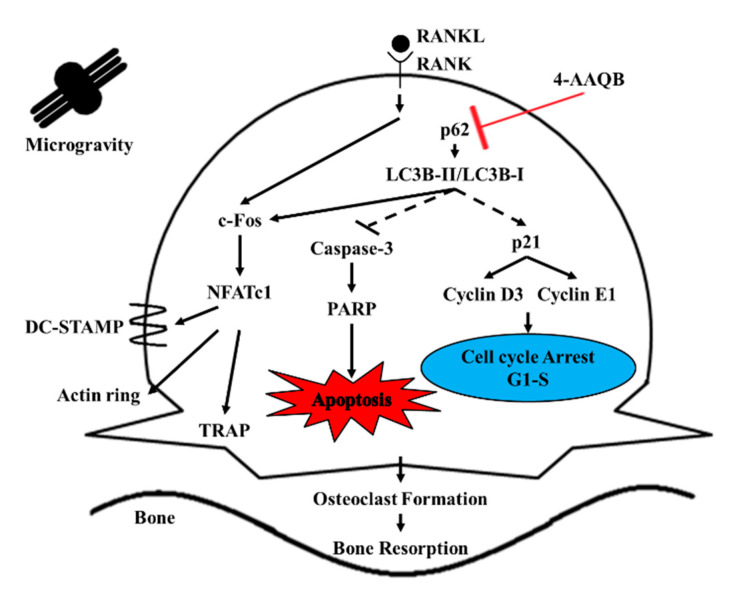
Proposed mechanisms of 4-AAQB in osteoclastogenesis.

4-AAQB, a bioactive compound from *Antrodia cinnamomea*, has shown anti-inflammatory and anti-hepatoma activities [26]. The present study aimed to investigate the inhibitory effects of 4-AAQB on osteoclastogenesis in both v-Xg and μXg conditions. We found that 4-AAQB alleviated osteoclast formation and function in both environments while 4-AAQB exerted a greater protective effect in μXg than in v-Xg. We further investigated the mechanistic impact of 4-AAQB under μXg conditions. 4-AAQB activated osteoclast apoptosis and caused cell cycle arrest accompanied by autophagy suppression (Figure 7). These results suggest that 4-AAQB inhibits osteoclastogenesis by regulating the autophagy pathway.

Excessive osteoclastogenesis is exhibited in μXg conditions [38,39]; this is the main reason for μXg-induced bone loss. Our study is the first to investigate the regulatory effects of 4-AAQB on osteoclastogenesis in both v-Xg and μXg conditions. We found that the effects of 4-AAQB in v-Xg were similar to those of ALN; however, in µXg, 4-AAQB performed more effectively than ALN. Our findings indicate that 4-AAQB exerted a better protective effect than ALN under μXg conditions, implying a potential role of 4-AAQB in μXg-induced bone loss. These results provide a basis for exploring the beneficial effects of 4-AAQB in vivo. The chemical structure of 4-AAQB is similar to coenzyme Q10 (CoQ10) [40]. Studies have shown that CoQ10 inhibits osteoclast differentiation by suppressing inflammatory responses, reactive oxygen species generation, and TRAP expression in vitro and in vivo [22,41]. In addition, CoQ10 induces the proliferation and differentiation of osteoblasts [42]. 4-AAQB might show similar mechanisms of action as CoQ10 in osteoblasts. However, this requires further investigation.

Targeting osteoclast apoptosis is a promising strategy for anti-osteoclastogenesis [43,44]. In addition to apoptosis, p21 controls cyclin-dependent kinases [45]. Cyclin D3 and cyclin E regulate the downstream pathways involved in osteoclast differentiation [46]. In our study, we showed that 4-AAQB activated apoptosis and caused cell cycle arrest at the G1-S phase, which is similar to the ALN mechanism [47,48]. Based on our findings, we suggest that 4-AAQB attenuates osteoclastogenesis by inducing cell apoptosis and causing cell cycle arrest at the G1-S phase. Interestingly, cell apoptosis is related to the autophagy pathway [32]. μXg has been reported to induce autophagy in pre-osteoclast RAW264.7 cells [49], which exerts a significant effect on osteoclast differentiation and function [50]. We showed that 4-AAQB suppressed autophagic flux in RANKL-treated cells, which is consistent with the findings of a previous study [27]. In addition, autophagy inhibitors 3-MA and CQ showed similar effects. These findings suggest that 4-AAQB attenuates osteoclast differentiation by suppressing autophagic flux. Autophagy also regulates osteoclast apoptosis [32] and cell cycle in osteoclast progenitors [51]. Combined with the results of apoptosis and cell cycle arrest, we suggest that 4-AAQB attenuates osteoclastogenesis primarily by inhibiting the autophagy pathway. It subsequently activates cell apoptosis and causes cell cycle arrest to alleviate osteoclast differentiation. Furthermore, in previous studies, autophagy was found to regulate cell apoptosis [52] and the cell cycle [53]. These signaling pathways have also been reported to participate in osteoclastogenesis [47,54]. Therefore, we concluded that 4-AAQB inhibits osteoclast differentiation by regulating autophagy to impact cell apoptosis and the cell cycle. Our findings were collected in simulated μXg conditions, not a real space environment, which is a limitation of this study. However, our results regarding the molecular mechanisms of 4-AAQB may be applied to understanding the space-induced bone loss of astronauts in the future.

## 5. Conclusions

4-AAQB attenuated μXg-induced osteoclastogenesis by suppressing autophagy, inducing apoptosis, and causing cell cycle arrest at the G1-S phase. Thus, we suggest that 4-AAQB may serve as a potential agent against μXg-induced osteoclast formation.
